# Substitutes for Lard and Olive Oil

**Published:** 1918-09-21

**Authors:** 


### NEW WAR EMERGENCY FORMULÆ.

### Substitutes for Lard and Olive Oil.

For the second time since the beginning of the war an alteration of several formulæ of the British Pharmacopœia has had to be made in consequence of the interruption of the free supply of certain ingredients. The first alteration became necessary because of the shortage of sugar and the commandeering of glycerin, and the second because of the restricted market for olive oil and lard. The new formulæ for preparations containing sugar and glycerin were referred to at the time they were introduced (see The Hospital, August 25, 1917, p. 414), and in the present article the essential details are given of the formulæ for compounds which formerly contained lard or olive oil. In passing it is not irrelevant to mention that in not a single instance has an official formula been changed because of the lack of an important medicinal agent; sugar and glycerin, lard and olive oil are useful as sweetening agents in the case of the two former, and as bases for ointments and liniments in the case of the two latter, but none of the four can be regarded as absolutely necessary for the treatment of disease; and, what is quite as much to the point, suitable substitutes for all of them are available. When we reflect that a large proportion, probably the majority, of the compounds of the Pharmacopœia contain ingredients which have to be brought across the seas —through the enemy's lines as it were—it is an eloquent tribute to our Navy that no essential medicinal agent has completely disappeared from the shelves of hospital dispensaries—at least we can think of none at the moment—and no alteration that really matters has had to be made in the composition of Pharmacopœial preparations.

The changes immediately under consideration were rendered necessary, as has already been intimated, by the shortage of lard and olive oil. In March of this year the General Medical Council gazetted an Order withdrawing from the Pharmacopœia four liniments and all the ointments in making which lard or suet was ordered to be used, and now, after a delay of six months, the Pharmaceutical Society has issued as an addendum to the British Pharmaceutical Codex a series of formulæ to take the place of those affected by the Medical Council's Order. The basis laid down for most of the ointments concerned is a substitute—Adeps Factitius—composed as follows:—

| | |
|---|---|
| Wool fat ... | 5 |
| Hard paraffin ... | 10 |
| Soft paraffin (white) ... | 85 |

This is suitable for use in making benzoated lard and the following ointments: aconitine, belladonna, capsicum, mercury, atropine, compound mercury, iodine, resin, cocaine, iodoform, and emollient ointment. Benzoated lard made from lard substitute is suitable for use in making the following ointments: cantharidin, gall, ammoniated mercury, red mercuric iodide, oleate of mercury, calomel, myrobalanes, lead iodide, potassium iodide, stavesacre, sulphur, and zinc.

Generally speaking, the new formulæ are quite simple; all the dispenser has to do in most cases is to use lard substitute instead of lard. Iodine ointment is one of the exceptions, since it formerly contained two proscribed ingredients—to wit, glycerine and lard. The new formula is as follows:—

| | |
|---|---|
| Iodine ... | 4 |
| Potassium iodide ... | 4 |
| Distilled water ... | 12 |
| Wool fat ... | 10 |
| Lard substitute ... | 70 |

The wool fat is melted in a water bath, the lard substitute is added, and the compound is then mixed with iodine, potassium iodide, and water previously triturated together in a glass or porcelain mortar.

The liniments affected are four in number, the chief being liniment of camphor, which enters into the composition of the three others—namely, liniment of chloroform, liniment of mercury, and liniment of turpentine and acetic acid. Liniment of camphor was formerly made with olive oil, and the substitute now prescribed is yellow liquid paraffin of a grade which answers to the tests definitely laid down. It is important that dispensers should obtain a yellow liquid paraffin of the designated quality, otherwise the camphor liniment produced will be unsatisfactory; in an inferior solvent the camphor will not remain in solution, and the liniment will also be unsightly in appearance. Sesame oil would no doubt have been better for the purpose, but this has not been prescribed because supplies are not freely available.

In addition to the preparations named there is one other for which a new formula is given—namely, compound solution of cresol. This was formerly made with castor oil, which, as everybody knows, is now wanted for a very important war purpose. In the new formula linseed oil is substituted, and the following is the method of manufacture:—

| | |
|---|---|
| Cresol (by weight) ... | 50.00 |
| Linseed oil (by weight) ... | 18.00 |
| Potassium hydroxide ... | 4.25 |
| Distillel water (by weight) to ... | 100.00 |

To the linseed oil at about 70° the potassium hydroxide (dissolved in 25 parts of distilled water at the same temperature) is added, thoroughly mixed and heated until a small portion of the resultant soap is found to dissolve in water without the separation of oily drops; the cresol is then added and mixed and the weight is adjusted by the evaporation or addition of distilled water.

These new formulæ are not official in that they have not been adopted by the General Medical Council, and inserted in the Pharmacopœia. Consequently dispensers are not legally compelled to use them, but in the absence of any other set of standards, and in view of the fact that these new formulæ have been well tested, it is to be hoped that they will be employed throughout the country, so that no matter at what pharmacy or what hospital a prescription may be dispensed a preparation of the same appearance and composition will be produced.

